# The relevance of spatial aggregation level and of applied methods in the analysis of geographical distribution of cancer mortality in mainland Portugal (2009–2013)

**DOI:** 10.1186/s12963-018-0164-6

**Published:** 2018-03-27

**Authors:** Rita Roquette, Baltazar Nunes, Marco Painho

**Affiliations:** 1 Nova IMS Information Management School, Lisbon, Portugal; 2 National Health Institute Doutor Ricardo Jorge, Avenida Padre Cruz, 1649-016 Lisbon, Portugal; 3 Escola Nacional de Saúde Pública, Lisbon, Portugal

**Keywords:** Spatial aggregation level, Cancer mortality, SMR, BYM, Portugal

## Abstract

**Background:**

Knowledge regarding the geographical distribution of diseases is essential in public health in order to define strategies to improve the health of populations and quality of life.

The present study aims to establish a methodology to choose a suitable geographic aggregation level of data and an appropriated method which allow us to analyze disease spatial patterns in mainland Portugal, avoiding the “small numbers problem.” Malignant cancer mortality data for 2009–2013 was used as a case study.

**Methods:**

To achieve our aims, we used official data regarding the mortality by all malignant cancer, between 2009 and 2013, and the mainland Portuguese resident population in 2011. Three different spatial aggregation levels were applied: Nomenclature of Territorial Units for Statistics, level III (28 areas), municipalities (278 areas), and parishes (4050 areas).

Standardized Mortality Ratio (SMR) and relative risk (RR) were computed with Besag, York and Mollié model (BYM) for the evaluation of geographic patterns of mortality data. We also estimated Global Moran’s I, Local Moran’s I, and posterior probability (PP) for the spatial cluster analysis.

**Results:**

Our results show that the occurrence of lower and higher extreme values of the standardized mortality ratio tend to increase with the decrease of data spatial aggregation. In addition, the number of local clusters is higher at small spatial aggregation levels, although the area of each cluster is generally smaller. Regarding global clustering, data forms clusters at all considered levels.

Relative risk (RR) computed by Besag, York and Mollié model, in turn, also shows different results at the municipalities and parishes levels. However, the difference is smaller than the difference obtained by SMR computation. This statement is supported by the coefficient variation values.

**Conclusions:**

Our findings show that the choice of spatial data aggregation level has high importance in the research results, as different aggregation levels can lead to distinct results.

In terms of the case study, we conclude that for the period of 2009–2013, cancer mortality in mainland Portugal formed clusters. The most suitable applicable spatial scale and method seemed to be at the municipalities level and Besag, York and Mollié model, respectively. However, further studies should be conducted in order to provide greater support to these results.

## Background

In the analysis of geographical distribution of any type of phenomena, particularly in the field of health, the spatial and temporal level of data aggregation is determinant. In fact, the aggregation of disease occurrence data affects the patterns of geographical distribution, as well as the analysis of potential factors that could promote its development.

In the analysis of health data, both spatial and temporal aggregated numbers are usually considered in order to preserve the individual’s confidentiality [1]. In many diseases, like cancer, the aggregation level tends to be higher, not only due to the needs of data confidentiality, but also to increase the rates’ statistical robustness.

However, as a result of the spatial aggregation of data, the number of geographical areas under analysis decreases and a reduction of geographical variation of disease patterns occurs. Thus, the aggregation of data is responsible for a decreased possibility of detecting clusters [2].

Conversely, when the spatial aggregation and population at risk are small, the rates’ statistical robustness tends to diminish. In these situations, higher values of the disease’s rate generally tend to cluster in areas with small populations [3]. This effect is known as the “small numbers problem.”

Consequently, it seems that there is a duality between aggregated data usage. On the one hand, disaggregated data are needed to obtain more accurate results in spatial data analysis. On the other hand, lower disaggregation tends to better preserve individual’s confidentiality and contribute to more robust rates. This issue is not actually recent [4], but it is still timely. There isn’t a common correct spatial scale to analyze all disease data types in different geographic locations. Thus, there remains the need to choose the most suitable spatial level of aggregation depending on the data under study.

Similarly, the choice of the most appropriate methods to fulfill the objectives is crucial to obtain more reliable findings. In cancer research, Standardized Mortality Ratio (SMR) is traditionally and widely used to calculate and map mortality data [5]. However, SMR may not be the best choice if the population dimension under analysis is small. Actually, the variability of rates tends to be more frequent in areas with small populations. As result, it can be difficult to separate real differences of rates from fluctuations due the rates’ instability [6].

One possible way to improve stability of small number rates could be using smoothing methods [7]. Among the methods applied to smooth SMR, the spatial autoregressive model Besag, York and Mollié (BYM) is commonly used [8].

The aim of this paper is to discuss a methodology which allows to choose a spatial aggregation level that suits the analysis of mortality data from all malignant cancer in mainland Portugal in 2009–2013. To achieve that, we: a) compared three different levels of spatial administrative units: Parishes, Municipalities and Nomenclature of Territorial Units for Statistics (level III); and b) we employed different methods of data analysis, namely, Standard Mortality Ratio (SMR), Global and Local Moran’s I, and Besag, York and Mollié Model.

## Methods

**Fig. 1:**
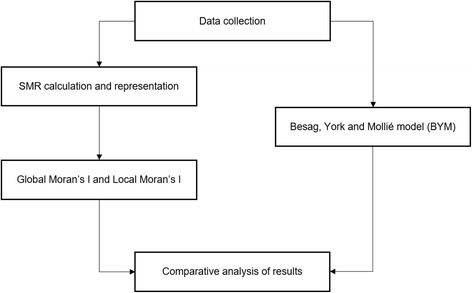
Schema showing the process of collection and analysis of mortality data by malignant neoplasm in mainland Portugal

Figure 1 presents a general view of the process of data collection and analysis. More details are described in the following sections.

After data collection we used two different methods to evaluate the geographical patterns of mortality by malignant cancer: SMR and BYM.

For cluster analysis we: a) applied Global and Local Moran’s I to SMR; and b) computed posterior probability (PP) of relative risk (RR) being higher than 1 to RR resulting from BYM.

Comparing the results obtained by different methods and geographical aggregation levels, we infer which of them is the most suitable to answer our research question. In the end we opted for the method and level which, on the one hand, ensures more statistical significance of the results, and on the other hand allows to clearly identify where high values of mortality form clusters.

Finally, in terms of software used*,* geographic computation was performed in ArcGIS 10.4 and R-INLA and the statistical analysis in IBM SPSS 22 and Excel 2016.

### Data collection

Data used was aggregated both spatial and temporally. Regarding spatial aggregation, we explored data in three hierarchically organized levels: parishes, municipalities, and Nomenclature of Territorial Units for Statistics (NUTS) III. Table 1 shows the descriptive statistics of the geographical units for each scale, for mainland Portugal.

**Table 1:** Descriptive statistics of geographical units of parishes, municipalities, and NUTS III at mainland Portugal in 2011

	Geographical units							Mean population
	n	Minimum (km2)	Maximum (km2)	Mean (km2)	Standard deviation	Quartile 1 (km2)	Quartile 3 (km2)	
Parishes	4050	0.05	435.31	22.00	35.39	5.14	23.00	2481
Municipalities	278	7.94	1720.60	320.46	283.60	134.42	399.52	36,142
NUTS III	28	814.58	8542.72	3181.75	2182.56	1563.12	4149.76	358,844

Parishes are the smallest administrative unit of Portugal, having a mean area of 22 km^2^ and a mean population (in 2011) of 2481 inhabitants. Municipalities are composed by parish sets, having a mean area and population of, respectively, 320 km^2^ and 36,142 inhabitants. NUTS III match with municipality sets and have a mean area of 3182 km^2^ and a mean population of 358,844 inhabitants. The NUTS III boundaries used correspond to the NUTS 2002 (PT) / NUTS 2003 (EU) delimitations, in force in all the years under study [9].

The administrative limits were collected from the Official Administrative Map of Portugal (CAOP), from 2011 [10], which is the central year of the analysis period (2009–2013). CAOP represents parish boundaries. We applied a geoprocessing method to transform parishes into municipalities and into NUTS III.

In terms of temporal aggregation, we considered mortality data over a five-year period, 2009–2013, given that it is recommended to use large populations, and data grouping in several years [11]. The central year, 2011, is the last census year, which allowed us to use more reliable data of risk population.

Both mortality and population data were obtained from Statistics Portugal (INE). We used codes C00 to C97 in data cancer collection, as defined in the 10th revision of International Statistical Classification of Diseases and Related Health Problems (ICD-10), and the resident population of Census 2011 in mainland Portugal.

### SMR calculation and representation

We decided to adopt SMR as one of the methods applied in data analysis because it is one of the most used methods in mortality data analysis, as mentioned [5]. Also, the major disadvantage of SMR is the comparison of areas with different population structure, which is not an issue in the case of our research [8].

SMR is the ratio between observed and expected cases of cancer deaths, enabling us to quantify differences in cancer mortality risks through all the study areas [12].

SMR (expressed in percentage) was computed by indirect method, as:

$$ SMR=\frac{O_j}{E_j}\ast 100 $$

Where:

O_j_ – Observed deaths in the geographical unit (j) in the period 2009–2013.

E_j_ – Expected deaths in the geographical unit (j) in the period 2009–2013, if mortality rate was the same of the reference population (P).

j – Geographical unit (each NUT III, municipality, or parish).

The formula applied to expected deaths was:

$$ {E}_j={\sum}_{i=1}^k\frac{D_i}{P_i}\ast {P}_{ij} $$

Where:

i – age group (1 to k).

D_i_ – Cancer’s deaths in age group (i) in mainland Portugal in the period 2009–2013.

P_i_ – Population of age group (i) in mainland Portugal at 2011, multiplied by 5.

P_ij_ – Population of age group (i) in geographical unit (j) at 2011, multiplied by 5.

As shown referred above, for the calculation of the reference population for the period 2009–2013, the population of 2011 was multiplied by five for years under analysis. Likewise, the population by age group and geographical unit (P_ij_) was computed using 2011 Census data multiplied by five. 18 age groups were considered: 0–4; 5–9; 10–14; 15–19; 20–24; 25–29; 30–34; 35–39; 40–44; 45–49; 50–54; 55–59; 60–64; 65–69; 70–74; 75–79; ≥ 80 years old.

The SMR distribution was represented in cartograms, all classified with the same legend, in order to allow for their comparison. Class selection is particularly difficult, because there are only 28 polygons in NUTS III and 4050 in parishes. So, we need to choose a number which allows us to identify patterns at NUTS III scale but which is also sufficiently detailed to parish patterns. As the ideal classification for all levels was impossible to achieve, we decided to adopt a classification previously used in literature for cancer’s SMR mapping [13]. Five class intervals were considered. The central class, 95–104%, represents the areas under analysis with values closer to the mainland value (corresponding to 100%). There are two classes with values below it (85–94%; < 85%) and two others above (105–114%; ≥115%).

In order to compare the three levels together, we represented SMR values in a boxplot and made graphics with SMR distribution in terms of population density.

The coefficient of variation (in percentage) of SMR was also calculated for each geographical unit under analysis, in order to allow us to compare the variation of values according to the geographical aggregation of the data. We applied the formula:

$$ {\mathrm{CV}}_{\mathrm{j}}=\frac{{\mathrm{SE}}_{\mathrm{j}}}{{\mathrm{SMR}}_{\mathrm{j}}}\ast 100 $$

Where:

SE_j_ – Standard Error of SMR in the geographical unit (J) in the period 2009–2013.

SMR_j_ – Standard Mortality Ratio in the geographical unit (J) in the period 2009–2013.

$$ \mathrm{And}\ {SE}_j=\frac{{\sqrt{O}}_j}{E_j} $$

O_j_ – Observed deaths in the geographical unit (j) in the period 2009–2013.

E_j_ – Expected deaths in the geographical unit (j) in the period 2009–2013, if mortality rate was the same of the reference population (p).

### Global Moran’s I and local Moran’s I

In the cluster analysis of SMR, both Global Moran’s I (Moran I) and Local Moran’s I (LISA) were applied [14]. The definition of neighborhood was made by the centroid distance method. Euclidean distances were measured and defined cutoffs that ensure that each area had at least one neighbor (a distance of 15 km to the parishes, 35 km to the municipalities, and 80 km to the NUTS III).

### Besag, York and Mollié model (BYM)

Relative risks (RR) and posterior probability (PP) were estimated by Besag, York and Molié (BYM) model, [15]. This model takes spatial homogeneity and heterogeneity into account [15].

BYM was computed to municipalities and parishes. It was not computed for NUTs III, because they have larger populations, the “small number problem” being absent.

Observed and expected cases of cancer and the neighbors’ matrix were considered. The neighborhood criterion adopted was contiguity of geographical units.

The same classes from SMR maps were adopted on RR maps for easier comparison.

We also computed the PP of RR > 1. We classified maps in five equal intervals, from 0 to 0.2 to 0.8–1.

Similar to SMR, descriptive statistics of relative risk were performed, as well as the computation of the coefficient of variation obtained by the formula:

$$ \mathrm{CV}=\frac{{\mathrm{SE}}_{\mathrm{j}}}{{\mathrm{RR}}_{\mathrm{j}}}\ast 100 $$

Where:

SE_j_ – Standard Error of RR in the geographical unit (j) in the period 2009–2013.

RR_j_ – RR in the geographical unit (j) in the period 2009–2013.

## Results

### SMR analysis

**Fig. 2:**
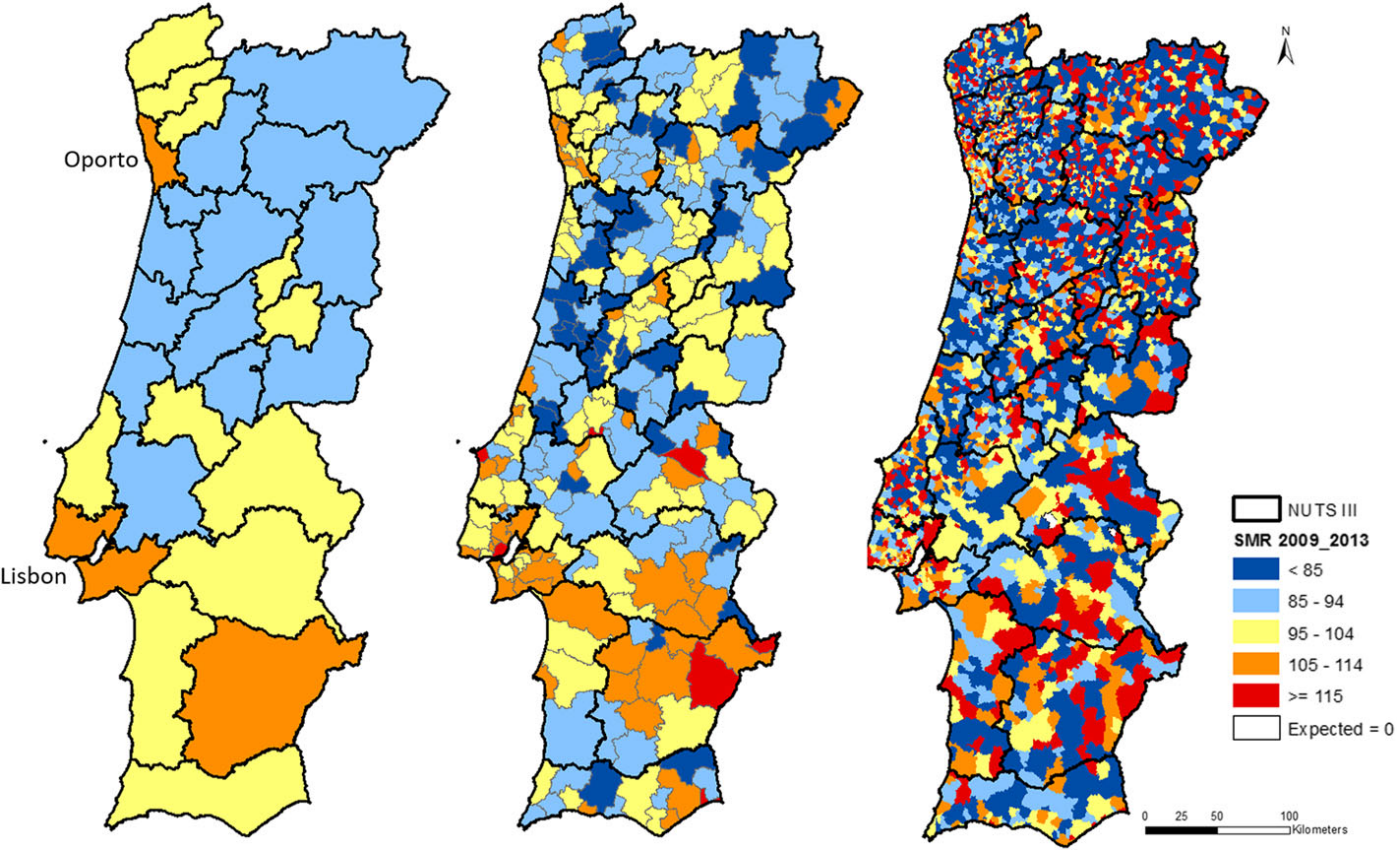
SMR by malignant neoplasm, standardized using indirect method by age group, in 2009–2013, by NUTS III (left map), municipalities (center map), and parishes (right map)

The distribution of SMR classes differs according to the spatial scale of data, as we can verify by comparison of maps by NUTS III, municipalities, and parishes presented in Fig. 2.

Maps showed the absence of extreme classes in NUTS III. In the other classes, the distribution patterns seemed homogeneous.

In the municipalities map, in turn, the occurrence of all classes spread throughout the map was observed, with a tendency of agglomeration of high values in the south. The higher values of SMR were greater than 130%, in two municipalities located in the southeast, on the border with Spain.

Concerning parishes, the different classes seemed to be randomly spread, originating a “salt-and-pepper-like effect” with no apparent patterns. The expected deaths by malignant neoplasm were null for two parishes (located in the south, close to the Spanish border), preventing the estimate of their SMRs. In the other 4048 parishes, SMRs varied between 0% and 400%.

Comparing polygon distribution by SMR class, for each of three spatial levels, we can emphasize that while the most represented class in municipalities was 85–94% (as in NUTS III), in parishes the most represented was of less than 85%, and both extreme classes together represented more than half of the polygons.

**Fig. 3:**
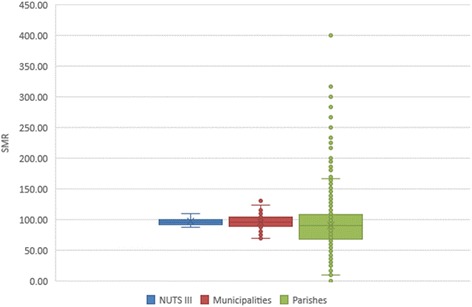
Boxplot of SMR, in the three different spatial aggregation levels

Descriptive statistics values, represented in the boxplots of Fig. 3, support what was said before. Boxplots show an increase of values range with the decrease of geographical unit level. At the NUTS III scale, the dispersion is very small. At parishes scale, there is a big dispersion of values, with the presence of many outliers.

Although there weren’t big differences in terms of mean (96.44 for NUTS III, 95.48 for municipalities, and 90.03 for parishes), in standard deviation, the differences are higher, increasing with the decrease of spatial aggregation of data (5.94 for NUTS III, 10.92 for municipalities, and 36.44 for parishes). Quartile 3 values present the same behavior (99.82 for NUTS III, 103.92 for municipalities, and 108.33 for parishes). In turn, quartile 1 has an inverse behavior (92.07 for NUTS III, 88.14 for municipalities, and 68.36 for parishes), which emphasizes the higher dispersion of values at small areas analysis.

**Fig. 4:**
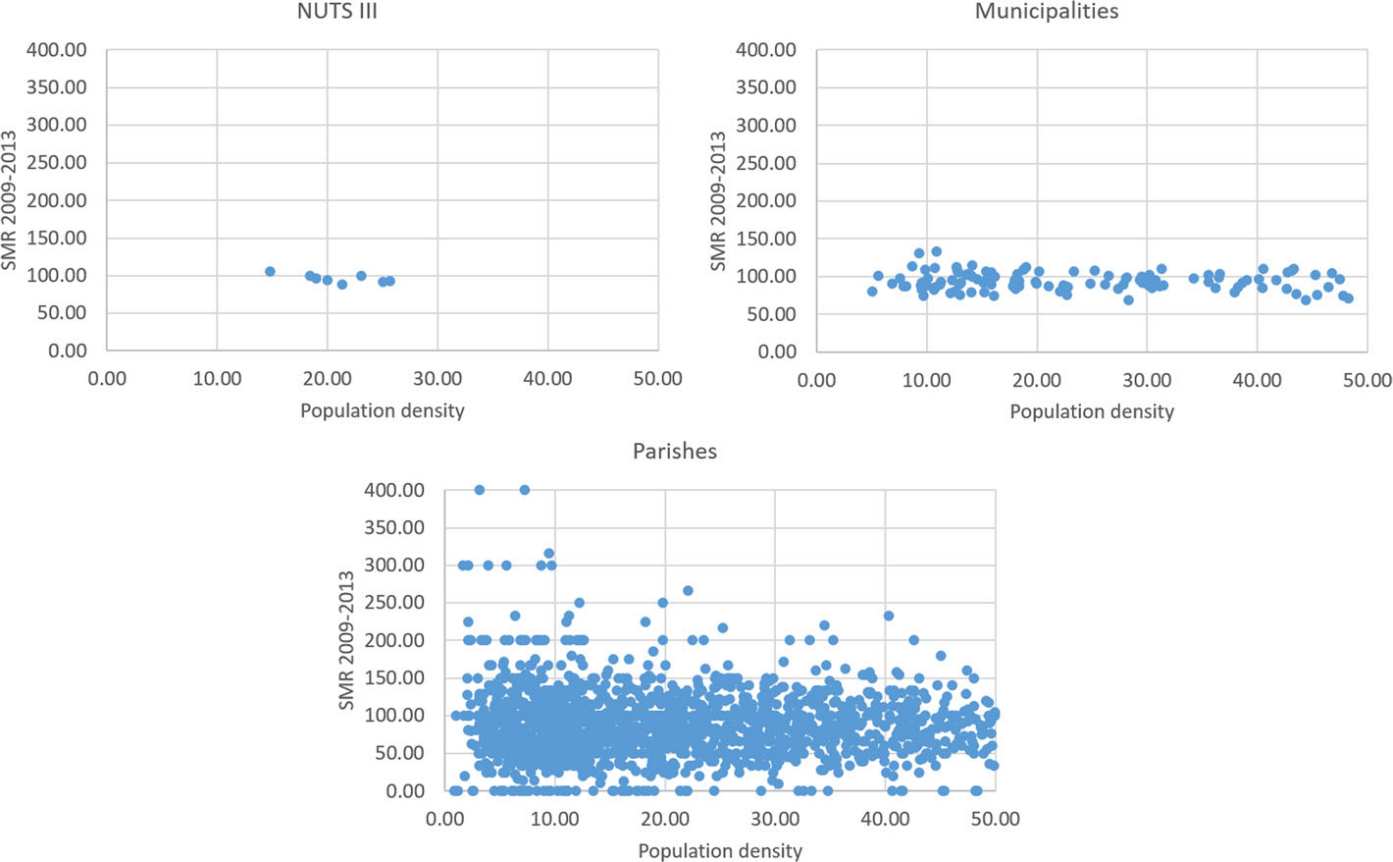
SMR values by population density, in geographical areas with population density ≤ 50 inhabitants/km^2^ in 2011

Regarding the effect of population density in rates, extreme values of SMR (such as 0 or 400) were verified in parishes which had low population density. In municipalities, in turn, two outliers were highlighted where SMR is higher than 130 and no values of 0. Finally, in NUTS III, there weren’t extreme values (Fig. 4).

In terms of significance, SMRs revealed to be statistically significant in 71.4% of NUTS III, 29.8% of municipalities, and 18% of parishes.

**Fig. 5:**
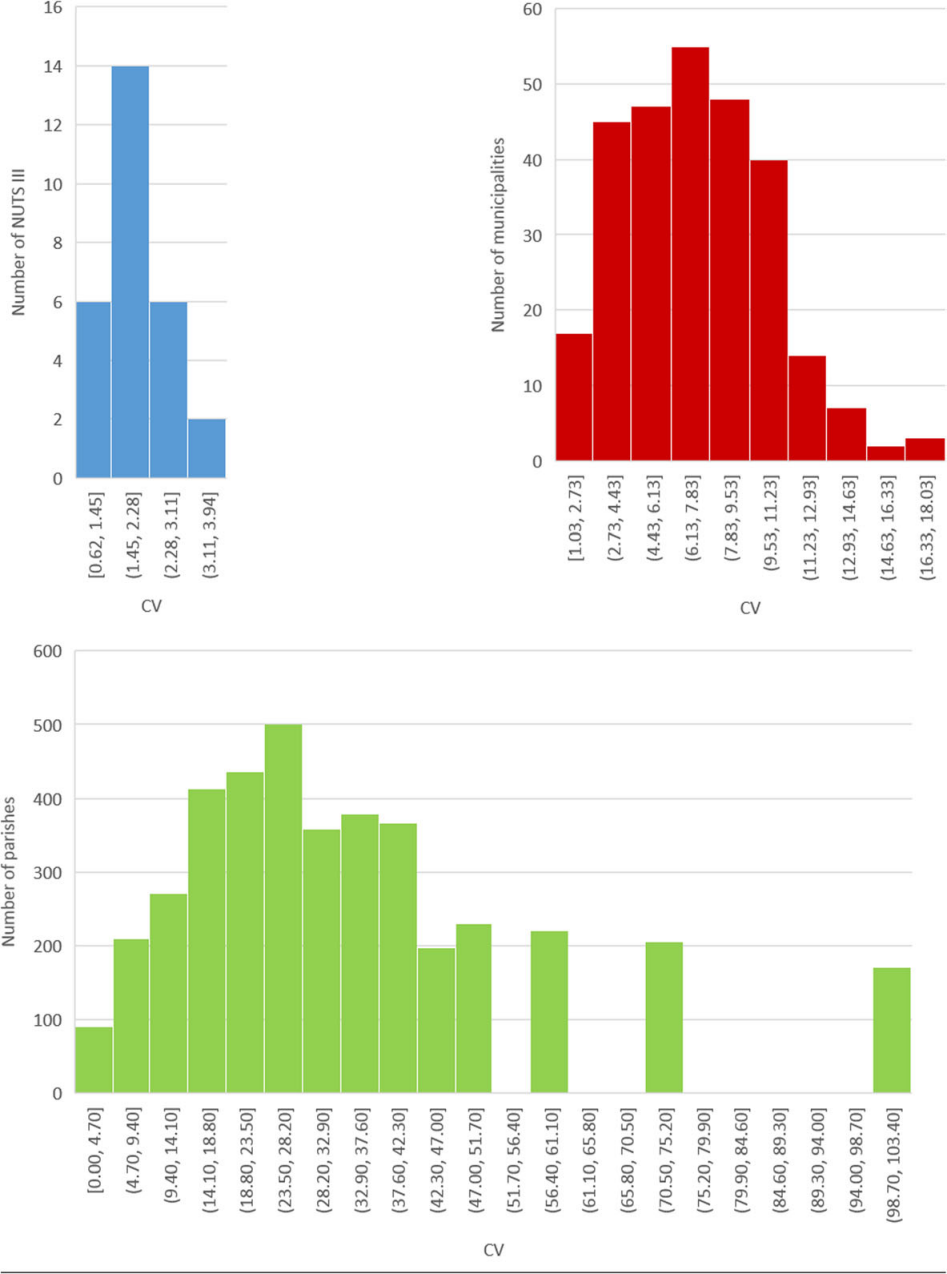
Coefficient of variation of SMR in NUTS III (top left), municipalities (top right), and parishes (bottom)

Coefficient of variation (CV) graphics, presented in Fig. 5, support these results. The interval of variation of CV values increased with spatial aggregation decreasing.

CV values were lower than 4% in NUTS III and 20% in municipalities. In parishes there was a higher variability of values, with a maximum near 100% and more than half of units with CV values higher than 20%.

### Moran’s I and LISA analysis

The Moran’s I result showed that there was spatial dependence in all analyzed levels. The higher the degree of spatial agglomeration, the higher the Moran I’s value (0.29 in NUT III, 0.22 in municipalities, and 0.03 in parishes).

**Fig. 6:**
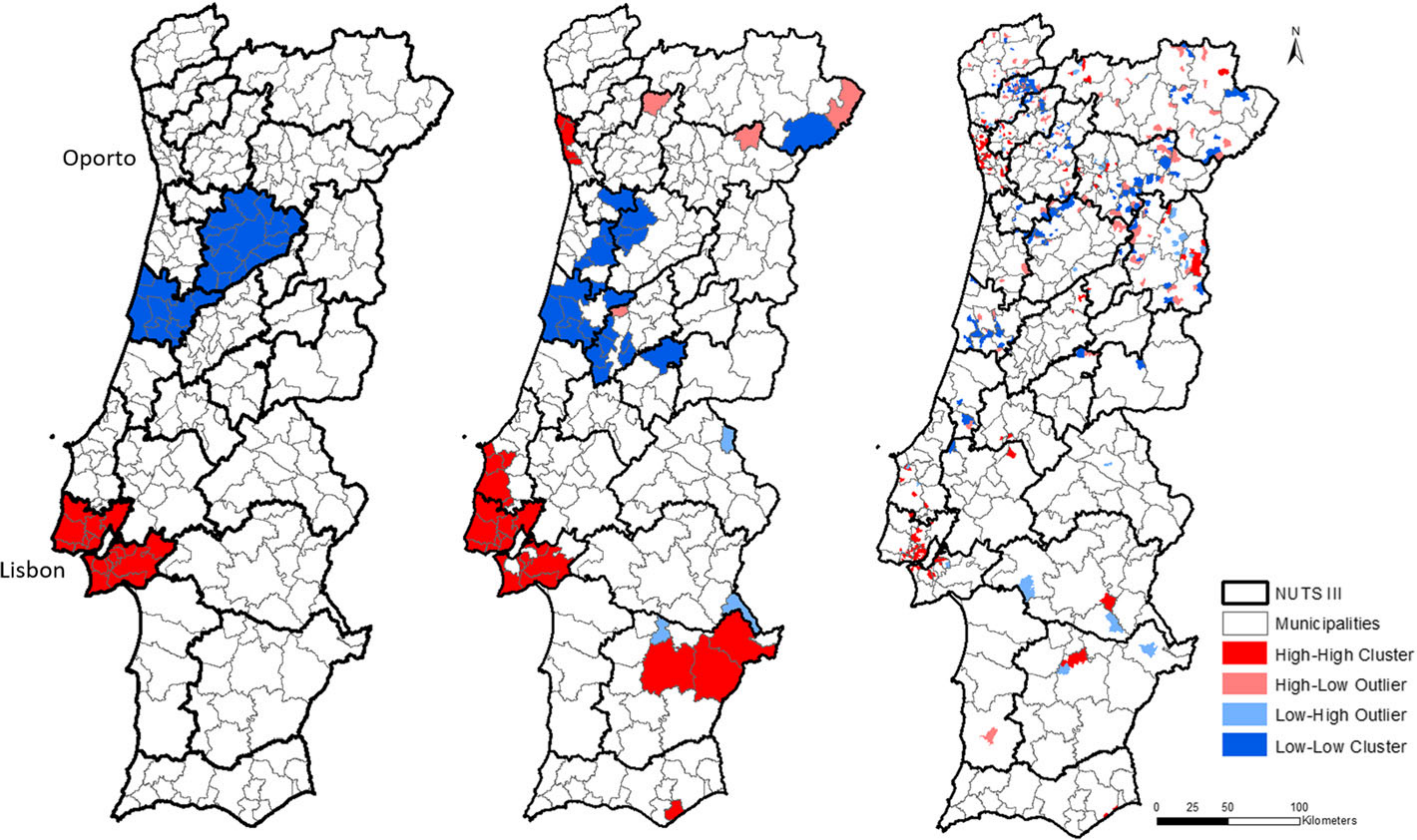
LISA map of SMR by malignant neoplasm, standardized by age group, in 2009–2013, by NUTS III (left map), municipalities (center map), and parishes (right map)

In turn, LISA cluster results were less consensual, as shown in Fig. 6.

The NUTS III map presented two High-High clusters, in the southwest coast, and two Low-Low clusters in the center.

The municipalities map, on the other hand, presented five High-High clusters, one composed of only one municipality, in the south and another in the southeast, two in the coastal southwest and finally, another cluster which included some municipalities in the coastal north. In the neighborhood of the southwest cluster there were two small High-Low clusters, which pointed to the possibility of the existence of outliers. There was another High-Low cluster, isolated, in the center east. There were also four Low-Low clusters, one in the north, and the other three in the northwest. It was also relevant to notice the two Low-High clusters, one joined to the North cluster and the other together with one of the other clusters. And another three single Low-High clusters, in the interior center and South.

Finally, in the parishes map sixty High-High clusters were identified (covering 145 parishes) and sixty-three Low-Low clusters (in a total of 181 parishes). There were also some High-Low and Low-High clusters.

### BYM analysis

**Fig. 7:**
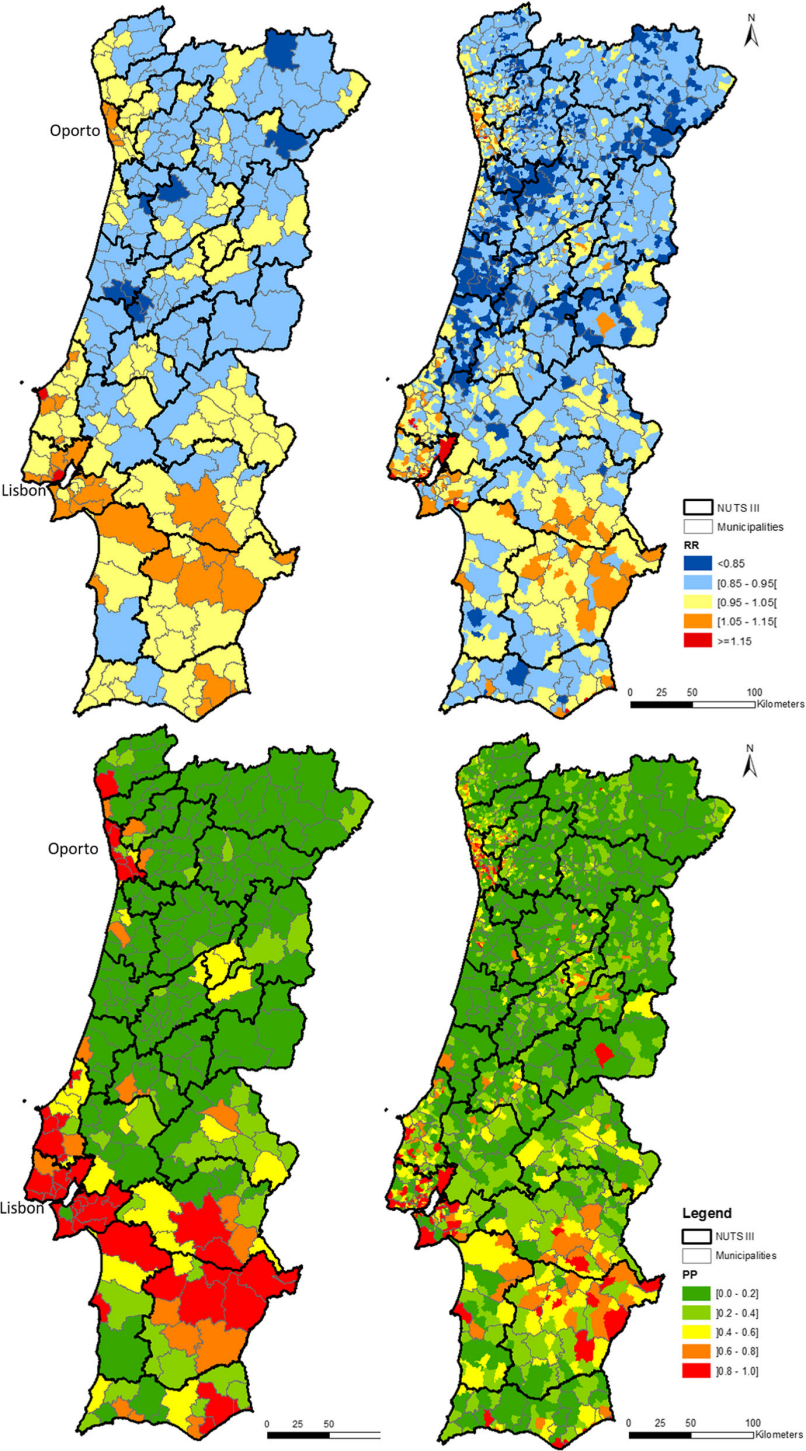
Relative risk (RR) of death by malignant neoplasm, standardized by age group, in 2009–2013, by municipalities (top left) and parishes (top right). Posterior probability of RR > 1, by municipalities (bottom left map) and parishes (bottom right)

The smoothing effect of the model was visible in RR maps on top left and right maps of Fig. 7.

In municipalities, there weren’t marked differences of classification between adjacent geographical areas. Although, relative risk exhibits a pattern of high values in a track which was extended from Lisbon to the southeast, in areas in the south and in coastal north. These patterns are supported by the map of PP (Fig. 7, bottom left), which presents similar patterns, composed by municipalities with PP > 0.8 of relative risk being higher than 1.

In turn, the distribution of high values of RR was more disperse in the parishes’ map than in the municipalities’ map. In addition, the PP of RR > 1 (Fig. 7, bottom right) was higher than 0.8 in only a few of these parishes.

**Fig. 8:**
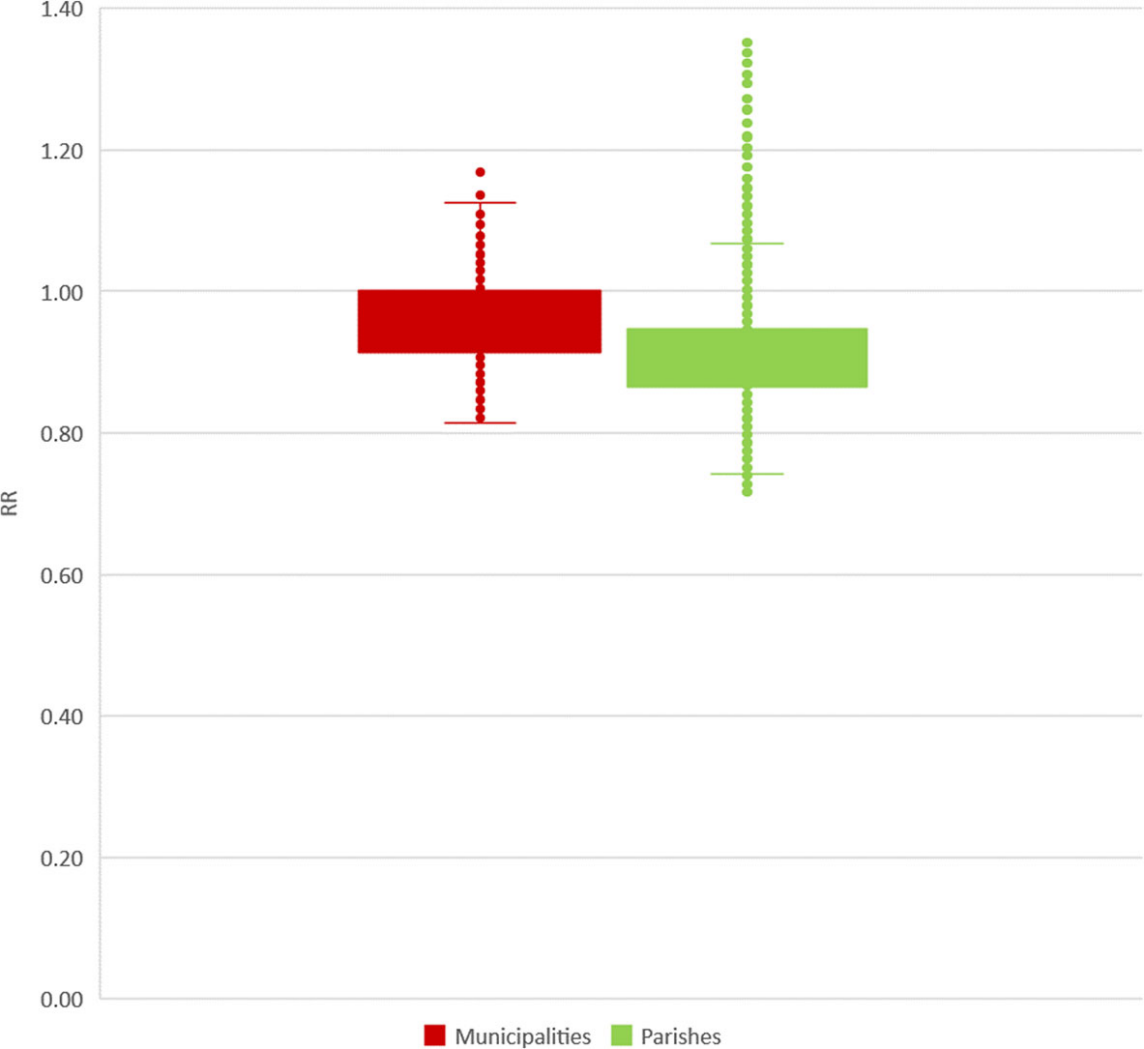
Boxplot of RR, in the three different spatial aggregation levels

These results were reflected in the boxplots of Fig. 8, which showed a higher number of outliers in parishes’ data than in municipalities’ data. Although mean values were similar (0.91 and 0.96, respectively) and standard deviation values equal (0.07), the minimum and maximum presented higher differences (respectively 0.72 and 1.35 for parishes and 0.82 and 1.18 for municipalities).

In terms of coefficient of variation of RR, this was also higher in parishes (8.32) than in municipalities (7.00), but lower than 9% in both levels.

**Fig. 9:**
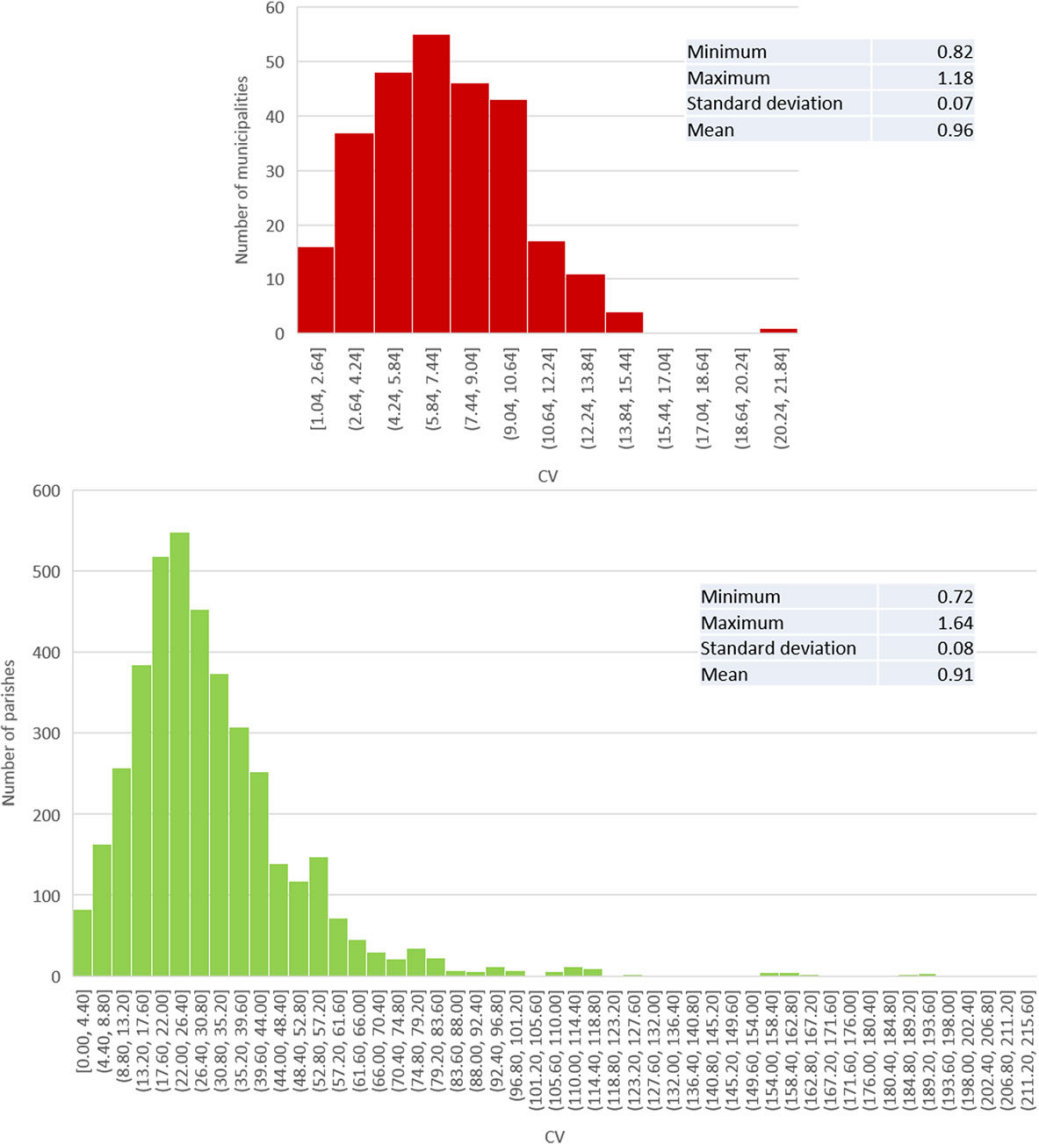
Coefficient of variation of RR in municipalities (top) and parishes (bottom)

The graphs of Fig. 9 showed the CV for each geographic unit under analysis. There wasn’t much CV variability in municipalities. In parishes, in turn, there were areas with higher CV, which point to rate instability.

## Discussion

We found that there are differences in cancer mortality geographical distribution according to the spatial level of aggregation of the data and the applied methods.

We notice that the most represented class of SMR is the same for almost all the levels of analysis. In fact, 85–94% is the predominant class in all considered levels, with the exception of the parishes (in which this is < 85%).

However, in comparing the proportion of extreme classes (< 85% and ≥ 115%), we point out that the unit percentage in these classes decreases with spatial bundling of data. These results are aligned with the knowledge that sparse populations can be associated with a rate’s extreme values [16].

We can also infer that larger geographic units under analysis tend to mask internal heterogeneity of data and that smooth mortality rates differ between areas [17].

In terms of statistical significance, we verified that the higher the data spatial aggregation, the greater the percentage of areas with statistically significant SMR values.

The coefficient of variation pattern is similar. Although all histograms presented in Fig. 5 have a left skewed curve, the interval of variation of CV values increase with the decrease of spatial aggregation. These results show the decrease of result homogeneity with the geographical units diminishing (with more than a half of parishes with CV > 20%) These results are according with Openshaw’s studies [18].

With regards to clustering, and based on Moran’s I results, we can affirm with 99% confidence that the distribution pattern of mortality by malignant cancer in mainland Portugal is clustered (for all spatial levels of analysis).

LISA results, in turn, differ according to spatial scale of analysis. In their evaluation it is important to take into account that a higher geographical aggregation corresponds to a lower capacity of cluster detection [2]. If data have a high spatial disaggregation, problems related with extreme rate values can appear, derived from small populations in geographical areas [19].

It is interesting to point out that the clusters of the parishes present some similarities with the municipalities, despite them being smaller than in the other levels. It should also be noted that there are clusters in the center east that there are not identified in the other levels.

It is also relevant to see if there are situations where a cluster in one scale corresponds to a cluster of the same type in all the related areas in a less aggregated scale. From the map analyses, we can see that both NUT III “Grande Lisboa” and all municipalities that compose it are classified as High-High clusters. In contrast, there is not any municipality belonging to a cluster (High-High or Low-Low) for which all the correspondent parishes are also integrated in a cluster.

The results discussed in the last paragraph could possibly be different if we had used the current delimitation of parishes. The Portuguese parish delimitation changed in 2013, and the 4050 parishes of mainland Portugal were rearranged to only 2882 [20]. However, we decided to not adopt that delimitation because our temporal period was previous to 2013.

Regarding BYM results, when compared to SMR maps, RR calculated by the BYM model has smoother patterns, both at municipality and parish scale, as to be expected [21]. The smoothing effect is stronger in the parishes map. In fact, there are few parishes classified in the higher class in the BYM map.

Similarly to what was described in SMR, CV values are higher at parish level. At mainland level, both municipalities and parishes register CV values indicating homogeneity of RR in the whole area. However, when we analyze CV values by each geographical unit, CV is higher than 20% in more than 70% of parishes.

Based on these results, it seems that municipalities are more suitable than parishes to analyze mortality by all cancers in mainland Portugal. This statement can be also considered if we want to investigate specific types of cancer, for which the number of cases will be lower.

In relation to applied methods, despite the smoothing effect, BYM results are according SMR and LISA maps. Particularly, we can highlight that the two areas classified in higher classes in the BYM municipalities map are also classified in this class in the SMR map.

In short, considering the results obtained by all considered methods at the municipalities scale, we can affirm that high values of mortality by all cancer types tend to agglomerate in a belt from Lisbon to the southeast and in the Oporto area (coastal north). At the parishes scale, SMR and LISA results are not so clear, but BYM results support what was said previously.

## Conclusions

Our study is based on mortality data by cancer as a whole, which seems to be a good basis for an exploratory analysis of the effect of spatial aggregation of data in the geographical patterns of this disease. We used a mix of methodologies – SMR rate, Moran’s I and LISA, and BYM – in order to understand the effect of spatial data aggregation in geographical patterns of cancer mortality in mainland Portugal.

Our results show that BYM has the power of smoothing rates and minimizes the possible random variation of rates [22], and simultaneously avoid the possible mask effect of inequalities, resulting from the adoption of large spatial scales [23]. In fact, our findings support that more spatial aggregated data leads to more reliable results but also contributes to a decrease of capacity to identify small (local) clusters, according to the literature [24].

There isn’t a proper scale for mapping disease. The scale depends on the occurrence of disease [5]. Based on our results, municipalities seem to be the best spatial level to answer the needs of power for cluster detection and reliability of estimated rates. Also, BYM seems to be more suitable than SMR to calculate and represent patterns of mortality by cancer in mainland Portugal.

Independently of spatial aggregation of data, our findings show that mortality by malignant neoplasm in mainland Portugal forms clusters. Previous studies had already made reference to differences in the geographical distribution of cancer mortality in Portugal [25]. This reinforces the necessity of continuing the studies in this area all the more because cancer and cardiovascular disease are responsible for more than 50% of the mortality burden in Portugal [26]. We think our work, and future work in this area, is very important to obtain up-to-date and reliable information that supports programs in the field of fighting cancer and research.

In summary, we think our work shows that the choice of spatial data aggregation has a high importance in research results, as different aggregation levels can lead to distinct results. Moreover, there is not a unique spatial aggregation or method suitable for one type of data or territory. So, in this type of research, it is very valuable to inspect different data aggregation levels and methods, in order to determine the most suitable for data under analysis.

## Abbreviations

BYM: Besag, York and Mollié model
CAOP: Official Administrative Map of Portugal
CV: Coefficient of variation
ICD-10: 10th revision of International Statistical Classification of Diseases and Related Health Problems
LISA: Local Moran’s I
Moran’s I: Global Moran’s I
NUTS III: Nomenclature of Territorial Units for Statistics (level III)
PP: Posterior probability
RR: Relative risk
SMR: Standardized mortality ratio

## References

[1] Bell BS, Hoskins RE, Pickle LW, Wartenberg D (2006). Current practices in spatial analysis of cancer data: mapping health statistics to inform policymakers and the public. Int J Health Geogr.

[2] Ozonoff A, Jeffery C, Manjourides J, White LF, Pagano M. Effect of spatial resolution on cluster detection: a simulation study. Int J Health Geogr. 2007;6:52. 10.1186/1476-072X-6-52 PMC2213641 18042281

[3] Pringle DG (1996). Mapping disease risk estimates based on small numbers: an assessment of empirical Bayes techniques. Economic and Social Review.

[4] Armstrong MP, Rushton G, Zimmerman DL (1999). Geographically masking health data to preserve confidentiality. Stat Med.

[5] Lawson AB. Statistical methods in spatial epidemiology. England: Wiley; 2013.

[6] Banerjee S (2016). Spatial Data Analysis. Annu Rev Public Health.

[7] Lawson AB, Browne WJ, CLV R. Disease mapping with WinBUGS and MLwiN. England: Wiley; 2003.

[8] López-Abente G, Aragonés N, García-Pérez J, Fernández-Navarro P (2014). Disease mapping and spatio-temporal analysis: importance of expected-case computation criteria. Geospat Health.

[9] INE (2015). NUTS 2013: As novas unidades territoriais para fins estatísticos.

[10] Carta Administrativa Oficial de Portugal (CAOP). versão 2011 [http://www.dgterritorio.pt/cartografia_e_geodesia/cartografia/carta_administrativa_oficial_de_portugal__caop_/caop__download_/carta_administrativa_oficial_de_portugal___versao_2011_2/]. Accessed 5 Feb 2017.

[11] Jensen OM. Cancer registration: principles and methods. France: IARC; 1991. 1894317

[12] Esteve J, Benhamou E, Raymond L. Statistical methods in cancer research. Descriptive epidemiology. IARC Sci Publ. 1994;4:1–302. 7698823

[13] Mota L, Falcão J (1997). 2° Atlas da mortalidade por cancro em Portugal 1990–1992.

[14] Anselin L (1995). Local indicators of spatial association—LISA. Geogr Anal.

[15] Besag J, York J, Mollié A (1991). Bayesian image restoration, with two applications in spatial statistics. Ann Inst Stat Math.

[16] McLaughlin CC, Boscoe FP (2007). Effects of randomization methods on statistical inference in disease cluster detection. Health & place.

[17] Tarkiainen L, Martikainen P, Laaksonen M, Leyland AH (2010). Comparing the effects of neighbourhood characteristics on all-cause mortality using two hierarchical areal units in the capital region of Helsinki. Health & place.

[18] Parenteau MP, Sawada MC. The modifiable areal unit problem (MAUP) in the relationship between exposure to NO2 and respiratory health. Int J Health Geogr. 2011;10:58. 10.1186/1476-072X-10-58 PMC3245430 22040001

[19] Goovaerts P (2008). Accounting for rate instability and spatial patterns in the boundary analysis of cancer mortality maps. Environ Ecol Stat.

[20] Lei n°11-A/2013, de 28 de Janeiro. In *Diário da República, Iª série, n°19*, vol. 11-A/2013; 2013. https://dre.pt/pesquisa/-/search/373798/details/maximized.

[21] Natário I (2005). Hierarchical Bayesian Models for Epidemiological Analysis of Rare Events Faculdade de Ciências da Universidade de Lisboa, Faculdade de Ciências.

[22] Santana P, Costa C, Mari-Dell'Olmo M, Gotsens M, Borrell C. Mortality, material deprivation and urbanization: exploring the social patterns of a metropolitan area. Int J Equity Health. 2015;14:55. 10.1186/s12939-015-0182-y PMC4483227 26051558

[23] Borrell C, Marí-Dell’Olmo M, Serral G, Martínez-Beneito M, Gotsens M (2010). Inequalities in mortality in small areas of eleven Spanish cities (the multicenter MEDEA project). Health & place.

[24] Gregorio DI, Dechello LM, Samociuk H, Kulldorff M (2005). Lumping or splitting: seeking the preferred areal unit for health geography studies. Int J Health Geogr.

[25] Pinheiro P, Tyczynski J, Bray F, Amado J, Matos E, Miranda A, Limbert E. Cancer in Portugal. In: IARC technical publication no 38. France: IARC; 2002. p. 72.

[26] Pereira M, Peleteiro B, Capewell S, Bennett K, Azevedo A, Lunet N (2012). Changing patterns of cardiovascular diseases and cancer mortality in Portugal, 1980–2010. BMC Public Health.

